# Anterior Sphincter-sparing Suturing of the Vesicourethral Anastomosis During Robotic-assisted Laparoscopic Radical Prostatectomy

**DOI:** 10.1016/j.euros.2023.04.007

**Published:** 2023-05-05

**Authors:** Luca Antonelli, Luca Afferi, Agostino Mattei, Christian Daniel Fankhauser

**Affiliations:** a Department of Urology, Luzerner Kantonsspital, Lucerne, Switzerland; b Department of Urology, Policlinico Umberto I, Rome, Italy; c University of Zurich, Zurich, Switzerland

**Keywords:** Prostate cancer, Robotic surgery, Minimally invasive surgery, Continence, Early recovery

## Abstract

**Background:**

Continence is an important functional outcome after robotic-assisted laparoscopic radical prostatectomy (RARP), and modifications of the surgical technique may improve outcomes.

**Objective:**

To illustrate a novel RARP technique and to describe the observed continence outcomes.

**Design, setting, and participants:**

A retrospective study of men treated with RARP between 2017 and 2021 was conducted.

**Surgical procedure:**

During RARP, periprostatic structures are preserved, the intraprostatic urethra is partially spared, and the anterior anastomosis stitches involve the plexus structures but not the anterior urethra.

**Measurements:**

A descriptive analysis of the pathological, functional, and short-term oncological outcomes was performed.

**Results and limitations:**

Of 640 men, 448 (70%) with at least 1 yr of follow-up and a median age of 66 yr were included. The median operative time was 270 min and the prostatic volume 52 ml. The transurethral catheter was removed after a median of 3 d, and leakage of urine in the first 24 h after catheter removal was observed in 66/448 patients (15%). Positive surgical margins were reported in 104/448 (23%). Prostate-specific antigen persistence after prostatectomy was observed in 26/448 (6%). During a median follow-up of 2 yr (interquartile range 1–3 yr), the biochemical recurrence after prostatectomy was observed in 19/448 patients (4%). One year after prostatectomy, 406/448 patients (91%) were continent and required no pad at all, while 42/448 (9%) required at least one pad per day.

**Conclusions:**

Not stitching the anterior urethra is a novel technical modification and may improve continence outcomes.

**Patient summary:**

We describe a novel way to stitch the bladder neck to the urethra after removal of the prostate using a surgical robotic system. Our technique appeared safe, with promising urinary continence results.

## Take Home Message

Sparing the bladder neck and the intraprostatic urethra, together with the preservation of the periprostatic structures and refraining from stitching the anterior urethra with the adjacent anterior part of the urinary sphincter, leads to favourable urinary continence after robotic-assisted laparoscopic radical prostatectomy.

## 1 Introduction

Robotic-assisted radical prostatectomy (RARP) is a treatment option for men with localised prostate cancer. Treatment of prostate cancer is challenging due to competing oncological and functional goals [1], [2], [3]. As these outcomes are influenced by the surgical technique, several modifications to RARP that aim to preserve and/or reconstruct the delicate periprostatic structures have been introduced [4]. We illustrate a novel end-to-end anastomosis technique of the vesicourethral anastomosis during which the anterior bladder neck is stitched to the venous plexus instead of the anterior urethra. This approach avoids direct injury or indirect tension to the anterior muscular part, which is the most important aspect of the sphincter.

## 2 Patients and methods

### 2.1 Informed consent

The study was approved by the Bioethics Committee (Swiss BASEC ID 2021-00181), and prior to surgery all patients signed an informed consent form to take part in this study. Patients were counselled about their diagnosis, prognosis, and different options for treatment specific to their disease, which may have included active surveillance, external beam radiotherapy, brachytherapy, and RARP. The expected benefits, risks, and likelihood of success for each option were discussed to ensure that the patient had sufficient understanding of the procedure and the potential risks specific to that individual. Surgical complications were discussed, including urinary incontinence, erectile or sexual dysfunction, nerve injury resulting in altered skin sensation or pain, infection, injury to vessels leading to bleeding or thrombosis, cancer recurrence, inguinal hernia, infertility, seroma, lymphedema, venous thromboembolic and arterial events, and general complications of anaesthetic. Patients were informed that any complication or disease recurrence may require further treatment.

### 2.2 Surgical procedure: patient positioning and docking

All procedures were performed by a single experienced surgeon (A.M.) with a four-arm da Vinci Si Surgical System (Intuitive Surgical-ISRG, Sunnyvale, CA, USA) as described previously [5], [6], [7]. In brief, during RARP, the patient is placed in a supine position, and a rectal catheter is introduced after general anaesthesia. The abdomen is shaved, prepped, and draped in a sterile manner. Firstly, a supraumbilical incision is made and a pneumoperitoneum of 15 mmHg is established using a Veress needle. The incision is widened, and an 8-mm robotic trocar (da Vinci System; Intuitive Surgical-ISRG) is introduced. The abdominal cavity is inspected with a 0° robotic laparoscope to exclude adhesions. Secondly, three additional 8-mm robotic trocars are placed under vision, followed by 12- and 5-mm assistant ports (Covidien, Dublin, Ireland) positioned cranially at two fingers’ breadth from the right robotic port to enable triangulation. Finally, the surgical table is tilted up 20–40° to reach the Trendelenburg position, and the robotic system is docked.

### 2.3 Surgical procedure: sphincter-sparing technique

**Fig. 1:**
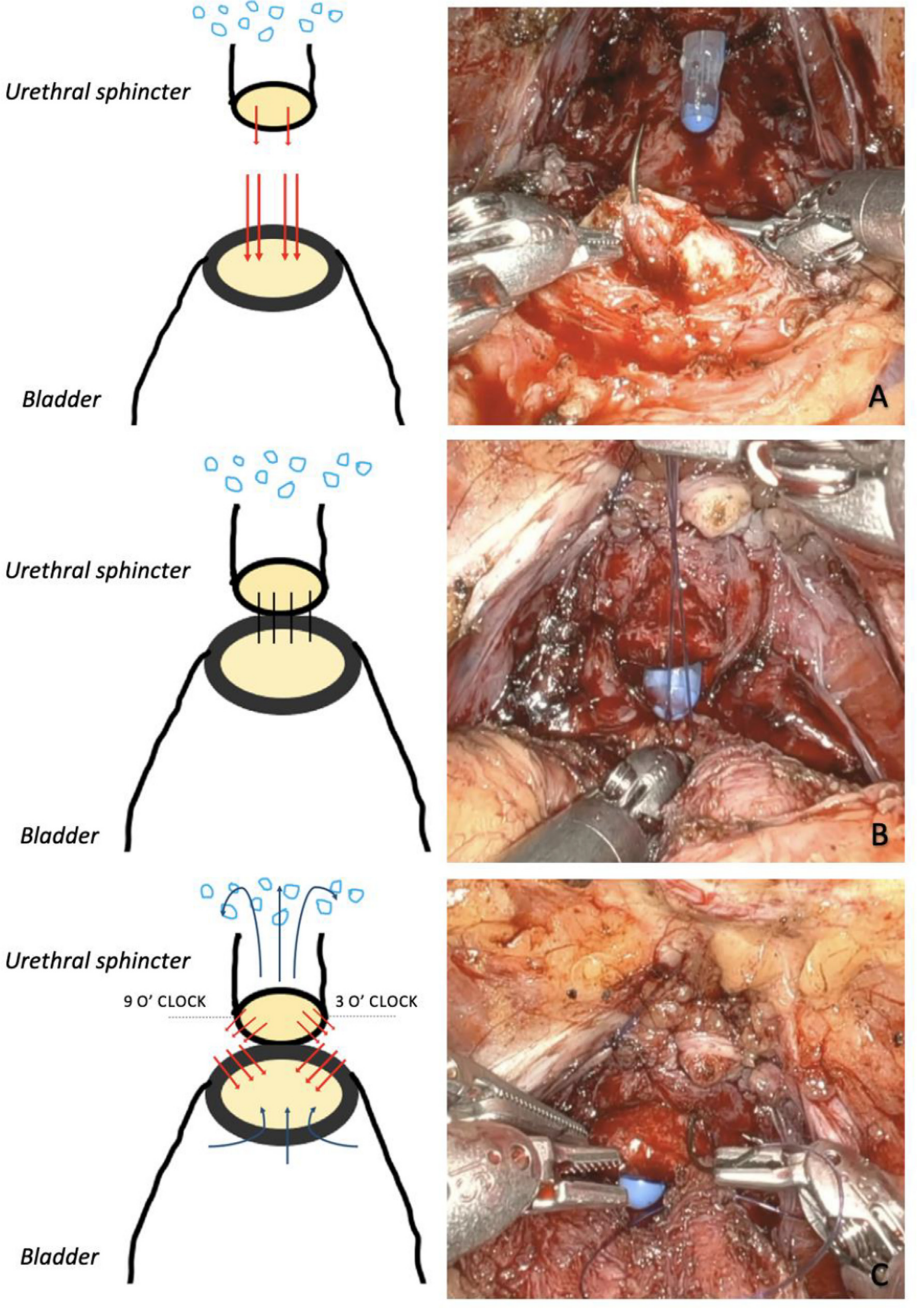
Surgical steps of the sphincter-sparing technique of the vesicourethral anastomosis. (A) First stiches of the vesicourethral anastomosis. (B) The bladder is slid down to the urethra. (C) Last stiches of the vesicourethral anastomosis, exclusively involving the venous plexus and not involving the urethra.

After sparing of the puboprostatic ligaments, the bladder neck, and as much functional length of the intraprostatic urethra, the vesicourethral anastomosis is created using two 3-0 Monocryl sutures, following the technique of Van Velthoven et al. [8]. The first stitches are placed at the 6 o’clock position on the posterolateral aspect of the bladder neck outside in and then through the urethra at the same location inside out and again outside in on the posterolateral aspect of the bladder **(**Fig. 1A). The bladder is slid down to the urethra in a parachute manner using the robotic needle holder (Fig. 1B). Proceeding anticlockwise, the running suture is placed three to five times more through both the bladder neck and the urethra in a similar fashion until the 3 o’clock position. The same is performed from 6 to 9 o’clock. Subsequent stitches from 9 to 12 o’clock and from 3 to 12 o’clock do not involve the urethra, but exclusively involve the already ligated venous plexus (Fig. 1C). A leak test is performed using 200 ml of saline water.

### 2.4 Surgical procedure: closure and dressings

No drains are used [9]. The prostate is removed through the widened supraumbilical incision. The fascia is closed with a 1-0 polyglactin suture, while all other incisions are closed with 3-0 polyglactin subcutaneous sutures. The skin is closed with staples (Appose ULC single-use skin stapler; Covidien) and covered with adhesive film dressings (Smith & Nephew, London, UK). The rectal catheter is removed.

### 2.5 Postoperative care

The patient can move, eat, and drink immediately after surgery. Our technique, which omits stitching of the anterior urethra, may lead to haematuria in some patients; this may require irrigation using a bladder syringe in selected cases. Thromboprophylaxis is prescribed once daily until discharge. Tadalafil (20 mg, twice weekly) is offered to all patients for penile rehabilitation during the first 12 wk after surgery.

### 2.6 Clinical follow-up

The patient is seen by the operating surgeon 10 d after surgery to remove staples and check wound conditions, and again 12 wk after surgery to assess functional results. Additional follow-up consultation is performed by the referring general practitioner or urologist. Prostate-specific antigen (PSA) measurements are recommended every 3 mo for 2 yr after surgery and every 6 mo for an additional 3 yr after surgery, after which yearly measurements are recommended. A biochemical recurrence has been defined as postoperative PSA >0.2 ng/ml.

### 2.7 Cohort study and video

All patients consented to the use of photographic and videographic material for educational purposes, in accordance with local institutional guidelines. Baseline variables included age, preoperative PSA value, preoperative prostate volume, and preoperative histology of the prostatic biopsy. Intraoperative variables included the duration of the procedure and the use of drainage. Operation time was defined from surgical sign-in to sign-out. Postoperative variables included the duration of catheter use, histology of the prostatectomy, surgical margin status, and biochemical recurrence. Follow-up was based on clinical notes and PSA values. Early continence was evaluated using pad tests [10]. All patients were asked to answer the expanded prostate cancer index EPIC-26 before surgery, and 3 and 12 mo after the surgery. Patient forms were submitted by mail. Missing continence data at 12 mo were supplemented by making individual phone calls. Urinary continence was defined as no pad use, whereas incontinence was defined as any amount of pad use, even the use of a safety pad [11]. All data were retrospectively analysed and reviewed. Statistical analyses were performed using R version 4.1.3 (R Foundation for Statistical Computing, Vienna, Austria).

## 3 Results

After the exclusion of 192 patients with a follow-up of <1 yr, 448 men treated between 2017 and 2021 with an RARP with the sphincter-sparing suture technique of the vesicourethral anastomosis were studied (Table 1). The median patient age was 66 yr (interquartile range [IQR] 61–71), the median preoperative PSA was 4.4 ng/ml (IQR 6.8–10.4), and the median prostate volume was 40 ml (IQR 30–50). Preoperative biopsy-proven histology included a Gleason score (GS) of 6 in 99 patients (22%), a GS of 7a in 191 patients (43%), a GS of 7b in 78 patients (17%), a GS of 8 in 55 patients (12%), a GS of 9 in 24 patients (5%), and a GS of 10 in one patient (<1%).

**Table 1:** Baseline characteristics of the included patients treated with the sphincter-sparing vesicourethral anastomosis suture technique

Number of patients	448
Age (yr), median (IQR)	66 (61–71)
Prostate volume (ml), median (IQR)	40 (30–50)
Preoperative Gleason score, *n* (%)	
6	99 (22)
7a	191 (43)
7b	78 (17)
8	55 (12)
9	24 (5)
10	1 (<1)
Preoperative PSA level (ng/ml), median (IQR)	4.4 (6.8–10.4)
Operative time (min), median (IQR)	270 (198–270)
Removal of catheter (median of PODs)	3
Median urine loss in the first 24 h from catheter removal in pad test	23 g/24 h (4–66)
Prostatectomy T stage, *n* (%)	
T2	293 (65)
T3a	85 (19)
T3b	70 (16)
Prostatectomy Gleason score, *n* (%)	
6	35 (8)
7a	227 (51)
7b	123 (27)
8	32 (7)
9	30 (7)
10	1 (<1)
Surgical margin status, *n* (%)	
R0	344 (80)
R1	104 (23)
Biochemical recurrence at 2-yr follow-up, *n* (%)	19 (4)

IQR = interquartile range; PODs = postoperative days; PSA = prostate-specific antigen.

The median operative time was 270 min (IQR 198–270). The transurethral catheter was removed a median of 3 d after RARP, and leakage of urine in the first 24 h after catheter removal was observed in 66/448 (15%) patients. In the first 24 h after catheter removal, the median urine loss in pad tests was 23 g/24 h (IQR 4–66).

The histology of prostatectomy specimens confirmed pT2a in 39 patients (9%), pT2b in six patients (1%), pT2c in 248 patients (55%), pT3a in 85 patients (19%), and pT3b in 70 patients (16%), as well as GS 6 in 35 patients (8%), GS 7a in 227 patients (51%), GS 7b in 123 patients (27%), GS 8 in 32 patients (7%), GS 9 in 30 patients (7%), and GS 10 in one patient (<1%). In 104 men (23%), a positive surgical margin was reported. During a median follow-up time of 2 yr, 19 patients (4%) experienced a biochemical recurrence.

One year after prostatectomy, 406/448 (91%) patients were continent, requiring no pad at all, while 42/448 (9%) required at least one pad per day. No interventions because of retention or stricture were reported.

## 4 Discussion

The most common side effect following RARP is urinary incontinence, which occurred in 74% of participants in the PROTECT study, a prospective randomised trial in the UK [12]. Similarly, results from the Scandinavian Prostatic Cancer Group Study Number 4 described urinary incontinence in 43% of patients (defined as any use of protection aid) 1 yr after surgery [13]. These results contrast with single-institution cohort studies of high-volume centres, which have reported urinary incontinence in as few as 11% of participants 3 yr after RARP [14]. These differences may be caused by patient selection, differences in outcome definitions, and/or differences in surgical technique.

The refinements of the surgical techniques during RARP may partially explain the divergent results in the literature regarding postoperative complications, including urinary incontinence. Since the introduction of RARP in 2001, the improved visualisation of pelvic structures has led to numerous technical modifications, to either preserve and/or reconstruct important anatomical structures [4]. Preservation includes the periprostatic structures [15], [16], neurovascular bundle [17], [18], bladder neck [19], [20], [21], [22], and urethral length [23], [24], [25], whereas reconstruction includes the bladder neck [26], [27], [28], [29] or periurethral structures [27], [28], [29].

In this study, we propose a further modification to the surgical technique by suturing the venous plexus instead of the anterior urethra, thereby sparing the urinary sphincter in this area. This technique may share some similarities with a periurethral suspension stitch [30], but it differs from other technical modifications, such as reconstruction of the posterior musculofascial [31], [32], [33] and/or anterior part of the vesicourethral anastomosis [34], Retzius-sparing approach [35], and the technique of suturing only the internal layer of the urethra without involving the outer rhabdosphincter [36]. Our technique not only spares the anterior internal layer, but also reduces tension on the sphincter by avoiding anterior stitches.

Our modification is based on the anatomical structure of the urethral sphincter, which has a circular horseshoe or omega shape, with more urinary sphincter fibres on the anterior aspect and fewer muscular fibres on the posterior aspect that end dorsally in the fibrous tendon [4], [37]. This suggests that the anterior part of the urethra plays a more critical role in urinary continence mechanisms. Although our continence outcome showed that over 90% of patients required no pads at all after 1 yr of follow-up, the lack of a randomised control arm makes it challenging to quantify the influence of this surgical modification. The presented technique suggests early recovery of urinary continence as only 15% of our patients demonstrated any measurable urine leakage in the 24 h after catheter removal, which seems to be a significant improvement compared with previous cohorts [38], [39], [40], [41].

Our technique and description of outcomes have some limitations. Like any new surgical modification, our technique requires a certain learning curve. Additionally, the follow-up data, which were partly collected through phone interviews, may be affected by biases from either the respondent or the interviewer. Ideally, this new technical modification of the surgical technique should be evaluated within a prospective trial with a comparative arm that includes random allocation. Furthermore, in patients with apical prostate cancer, valid concerns have been raised regarding whether prostatic urethral preservation may compromise oncological outcomes [25].

## 5 Conclusions

We demonstrated a novel approach to suture the vesicourethral anastomosis during robotic-assisted laparoscopic radical prostatectomy that spares the anterior urethra and demonstrates favourable urinary continence results. Ideally a multi-institutional randomised trial with longer follow-up would be performed to properly define the exact benefits of this modification.

  ***Author contributions:*** Christian Daniel Fankhauser had full access to all the data in the study and takes responsibility for the integrity of the data and the accuracy of the data analysis.

  *Study concept and design*: Fankhauser.

*Acquisition of data*: Afferi.

*Analysis and interpretation of data*: Antonelli.

*Drafting of the manuscript*: Antonelli, Fankhauser.

*Critical revision of the manuscript for important intellectual content*: Fankhauser, Mattei.

*Statistical analysis*: Antonelli, Fankhauser.

*Obtaining funding*: None.

*Administrative, technical, or material support*: None.

*Supervision*: Mattei.

*Other*: None.

  ***Financial disclosures:*** Christian Daniel Fankhauser certifies that all conflicts of interest, including specific financial interests and relationships and affiliations relevant to the subject matter or materials discussed in the manuscript (eg, employment/affiliation, grants or funding, consultancies, honoraria, stock ownership or options, expert testimony, royalties, or patents filed, received, or pending), are the following: None.

  ***Funding/Support and role of the sponsor:*** None.
